# Comparison of multiparametric MRI‐based and transrectal ultrasound‐based preplans with intraoperative ultrasound‐based planning for low dose rate interstitial prostate seed implantation

**DOI:** 10.1002/acm2.12592

**Published:** 2019-04-19

**Authors:** Elisha T. Fredman, Bryan J. Traughber, Andrew Gross, Tarun Podder, Valdir Colussi, Robert Vinkler, Mitchell Machtay, Rodney J. Ellis

**Affiliations:** ^1^ Department of Radiation Oncology University Hospitals Seidman Cancer Center Cleveland Medical Center Case Western Reserve University Cleveland OH USA; ^2^ Division of Radiation Oncology Louis Stokes Cleveland VA Medical Center Cleveland OH USA; ^3^ Northeast Ohio Medical University Rootstown OH USA

**Keywords:** brachytherapy, LDR, MRI, multiparametric, prostate, TRUS

## Abstract

**Purpose:**

Transrectal ultrasound images are routinely acquired for low dose rate (LDR) prostate brachytherapy dosimetric preplanning (pTRUS), although diagnostic multiparametric magnetic resonance imaging (mpMRI) may serve this purpose as well. We compared the predictive abilities of TRUS vs MRI relative to intraoperative TRUS (iTRUS) to assess the role of mpMRI in brachytherapy preplanning.

**Materials and methods:**

Retrospective analysis was performed on 32 patients who underwent iTRUS‐guided prostate LDR brachytherapy as either mono‐ or combination therapy. 56.3% had pTRUS‐only volume studies and 43.7% had both 3T‐mpMRI and pTRUS preplanning. MRI was used for preplanning and its image fusion with iTRUS was also used for intraoperative guidance of seed placement. Differences in gland volume, seed number, and activity and procedure time were examined, as well as the identification of lesions suspicious for tumor foci. Pearson correlation coefficient and Fisher's Z test were used to estimate associations between continuous measures.

**Results:**

There was good correlation of planning volumes between iTRUS and either pTRUS or MRI (*r* = 0.89, *r* = 0.77), not impacted by the addition of hormonal therapy (*P* = 0.65, *P* = 0.33). Both consistently predicted intraoperative seed number (*r* = 0.87, *r* = 0.86). MRI/TRUS fusion did not significantly increase surgical or anesthesia time (*P* = 0.10, *P* = 0.46). mpMRI revealed suspicious focal lesions in 11 of 14 cases not visible on pTRUS, that when correlated with histopathology, were incorporated into the plan.

**Conclusions:**

Relative to pTRUS, MRI yielded reliable preplanning measures, supporting the role of MRI‐only LDR treatment planning. mpMRI carries numerous diagnostic, staging and preplanning advantages that facilitate better patient selection and delivery of novel dose escalation and targeted therapy, with no additional surgical or anesthesia time. Prospective studies assessing its impact on treatment planning and delivery can serve to establish mpMRI as the standard of care in LDR prostate brachytherapy planning.

## 1 INTRODUCTION

The use of transrectal ultrasound (TRUS)‐based treatment planning for prostate seed implantation has been standard practice over the past two decades, supported by the Radiation Therapy Oncology Group study RTOG 98‐05. In this multicenter phase II trial, patients with localized prostate adenocarcinoma (PCa) underwent a preplanning TRUS (pTRUS) volume study alone to plan and guide transperineal low dose rate (LDR) permanent seed implant procedures. This study showed good biochemical control rates, favorable toxicity profiles, and overall survival comparable to other brachytherapy, external beam, and surgical series.^1^, ^2^ In addition to preplan imaging which is critical for determining the correct seed quantity and activity, intraoperative planning using TRUS (iTRUS) has been shown to provide important, accurate volume and mapping information to enhance seed placement and limit toxicity to nearby organs.^3^, ^4^

Application of magnetic resonance imaging (MRI) for identification and diagnosis of PCa dates back over 30 years.^5^ While older iterations of prostate MRI technique lacked sensitivity and specificity (particularly for early‐stage tumors),^6^ MRI performance has rapidly improved as higher resolution imaging has evolved over the past decade and is expected to further improve with sequence optimizations and other 3D resolution applications. In this setting, MRI has emerged as a useful tool for assessing preoperative staging of PCa.

Most recently, multiparametric MRI (mpMRI) sequences (T2‐weighted, diffusion‐weighted, DCE, MR spectroscopic imaging) have been shown to add important functional data to standard cross‐sectional findings, motivating the European Society of Urogenital Radiology (ESUR) to publish clinical guidelines for its use in PCa detection and staging.^7^, ^8^ Beyond its diagnostic utility, strong evidence is evolving for the role of mpMRI and image fusion as a useful aid in the treatment planning of prostate cancer.^9^ mpMRI in addition to TRUS may enable more precise targeting of high‐risk intraprostatic regions without unnecessarily increasing dose to surrounding structures, thereby improving local control.^10^, ^11^

In this new era of pretreatment MRI volume studies, the standard pTRUS, which causes a fair amount of patient discomfort and requires additional time and staff, may prove to be redundant. Concern, however, has been raised over the consistency between prostate volumes measured on TRUS compared to those from MRI. While some reports have demonstrated a tendency of preplan MRI (as well as CT) to overestimate prostate volume compared with ultrasound,^12^, ^13^ others found MRI to underestimate gland size relative to TRUS.^14^, ^15^

In order to further study the ability of MRI‐based preplanning to reliably predict the intraoperative TRUS‐based parameters for LDR brachytherapy, we prospectively performed a series of dosimetric preplans using both pTRUs and mpMRI during our transition from pTRUS‐ to mpMRI‐based planning. Specifically, we compared both pTRUS‐only–planned studies and MRI‐planned studies with a final iTRUS plan to determine the frequency and magnitude of dosimetric changes. The impact of volumetric variation was quantified through changes in the total seed activity necessary and, therefore, in the number of seeds required. The burden of MRI/TRUS fusion was assessed through changes in total procedure and anesthesia time.

## 2 METHODS

Institutional Review Board (IRB) approval was obtained for a retrospective review of our prospectively planned patients with localized prostate cancer treated who underwent LDR permanent seed implantation at our institution from September 5, 2012 to September 6, 2013. Thirty‐two patients underwent LDR permanent seed implantation during the study period, all of whom received a pTRUS volume study from which the quantity of seeds and total activity were determined. During the transition to MRI‐based preplanning, an additional mpMRI was performed on 14 of these patients (43.7%). All preplan imaging was acquired in the department of radiation oncology, overseen by a single radiation oncologist who specializes in brachytherapy. The same radiation oncologist, assisted by a single certified medical physicist, performed the brachytherapy preplanning, iTRUS planning, and seed implantation.

Pretreatment volume studies were acquired 2–3 weeks prior to the brachytherapy procedure. pTRUS was performed using a standard rectal ultrasound probe (BK Medical, Model: Flex Focus 8848) mounted on a manual stepper unit and template, synchronized with MIM symphony software/planning system (MIM, Beachwood, OH). Patients were placed in the dorsal lithotomy position and pTRUS images were acquired at 5 mm spacing. These cross‐sectional images were used to delineate the required contours and generate a pTRUS‐based dosimetric plan. For treatment planning with MRI, multiparametric sequences (T1, T2, dynamic contrast‐enhanced series (DCE), diffusion‐weighted imaging (DWI)) were acquired through the pelvis with patients in the supine position, and the T2 sequence was used to derive the prostate volume and the number of brachytherapy seeds, from which total seed activity was determined. Multiparametric sequences were used to radiographically identify regions highly suspicious for tumor foci based on the Prostate Imaging Reporting and Data System (PIRADS) scoring system.^8^ Typically, a combination of the T2‐weighted series then verified on DCE and DWI were referenced to identify foci of disease.

Treatment planning utilizing mpMRI was conducted using MIM planning software to facilitate automated target and normal tissue volume transfers from diagnostic MRI planning to the brachytherapy MIM Symphony program in conjunction with TRUS imaging. Automated MRI‐US rigid co‐registration, with an estimated 1–2 mm‐associated registration error, allowed for more accurate intraoperative adjustments from the initial volume study to further refine treatment dosimetry. A manual readjustment/alignment was also at the operator's disposal to improve fusion accuracy.

Implants as monotherapy were performed using either ^125^I or ^103^Pd isotopes (145 Gy and 125 Gy, respectively), with activities of 2.33U/1.80 mCi per seed for ^103^Pd, and 0.51U/0.40 mCi per seed for ^125^I. An automated planning target volume (PTV) was generated as an expansion of 2 mm in the axial directions, excluding posteriorly to avoid the rectal interface, and an expansion of 5 mm was performed in the craniocaudal directions. A peripheral loading technique was applied in order to generate a relatively homogenous dose cloud of 99% of prescription dose covering the target. Preoperative planning parameters and dose constraints were as per institutional standard. When a high‐risk PIRADS 4 or 5 lesion was identified on mpMRI, the region was targeted for dose escalation to 200% of prescription. No targeted dose escalation could be attempted on cases planned with TRUS alone.

Just prior to the implant procedure, an iTRUS volume study was obtained to determine motion and change in organ dimensions since the original pretreatment imaging. Patients were placed in dorsal lithotomy with their feet in stirrups to allow approximately a 90° hip and knee flexion. An US probe identical to the one used for preplan TRUS was used to capture 5 mm images along the entire prostate superior–inferior axis. Using the MIM software, the radiation oncologist recontoured the prostate in the operating room. An intraoperative treatment plan was then created based on the new target volume, constituting the final treatment plan to guide seed placement and dose distribution. After treatment, a postoperative pelvic CT scan with 3 mm slice thickness was obtained for comparison to the final delivered plan. Volumes from preplan MRI and TRUS were compared with intraoperative volume studies. Additionally, final postoperative dosimetry from same‐day CT scan was compared with intraoperative and real‐time dosimetry during the procedure. Prostate volumes were contoured independently by the attending radiation oncologist and medical physicist and compared for a final consensus volume. The association between two continuous measures was estimated by Pearson correlation coefficient for the two imaging methods for agreement with the iTRUS treatment. The difference of two coefficients was examined by Fisher's *Z* transformation test. A *P*‐value of < 5% was considered statistically significant.

## 3 RESULTS

Patient demographics between pTRUS alone and pTRUS/mpMRI groups were comparable (Table 1). 88.9% and 78.6% of the pTRUS and mpMRI groups, respectively, had a pretreatment PSA < 10 ng/mL, 77.8%, and 71.4% had Gleason scores 6 or 7 and 88.9% and 85.7% had a clinical stage of T1c.

**Table 1:** Patient demographics (n = 32)

Parameter	Value (%)	
	mpMRI (n = 14)	pTRUS (n = 18)
Age	65.5 ± 7.4	61.8 ± 8.0
PSA (ng/mL)		
0–4	5 (35.7)	4 (22.2)
4.1–10	6 (42.9)	12 (66.7)
10.1–20	2 (14.3)	1(5.6)
>20	1 (7.1)	1 (5.6)
Gleason score		
≤6	3 (21.4)	4 (22.2)
7	7 (50.0)	10 (55.6)
8–10	4 (28.6)	4 (22.2)
Clinical T stage		
T1a‐c	12 (85.7)	16 (88.9)
T2a	0 (0)	0 (0)
≥T2b	2 (14.3)	2 (11.1)
NCCN risk group		
Low	2 (14.3)	3 (16.7)
Intermediate	7 (50.0)	11 (61.1)
High	5 (35.7)	4 (22.2)
Hormone Therapy	7 (50.0)	5 (27.8)

mpMRI, multiparametric magnetic resonance imaging; TRUS, transrectal ultrasound; PSA, prostate‐specific antigen.

**Figure 1:**
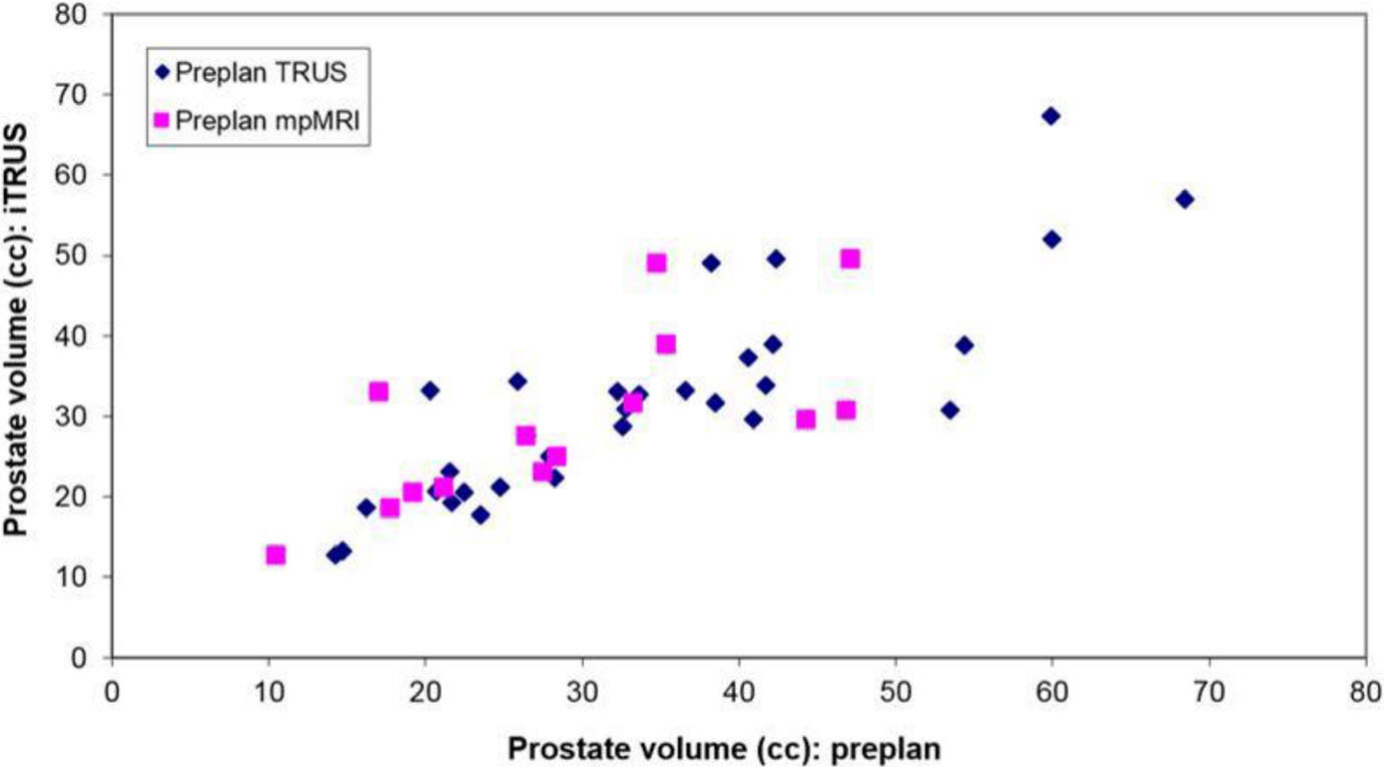
Pearson product‐moment correlation coefficient of iTRUS‐based prostate volume with pTRUS (*r* = 0.89) and mpMRI (*r* = 0.77).

Both pTRUS‐based and MRI‐based preplans accurately and consistently predicted the intraoperative prostate volume. The mean differences between iTRUS vs pTRUS and iTRUS vs MRI were 5 ± 4 cc (*P *=* *0.60) and 4 ± 4 cc (*P *=* *0.58), with correlation coefficient *r* values of 0.89 and 0.77, respectively (Fig. 1). The between sample difference was similarly nonsignificant (*P *=* *0.97). The mean percent differences between iTRUS vs pTRUS and iTRUS vs MRI were 15 ± 12% and 12 ± 11%, respectively. The predictive abilities of pTRUS and MRI were comparable as well in the subset of patients who had received prior HT, with mean differences between iTRUS vs pTRUS and iTRUS vs MRI of 4 ± 3 cc (*P *=* *0.65) and 4 ± 5 cc (*P *=* *0.33), respectively. The intersample difference was also nonsignificant (*P *=* *0.75). The mean percent differences between iTRUS vs pTRUS and iTRUS vs MRI were 12 ± 8% and 12 ± 7%, respectively. When stratifying for HT use, the difference in volume for the preplanning modalities was nonsignificant (*P *=* *0.35).

**Figure 2:**
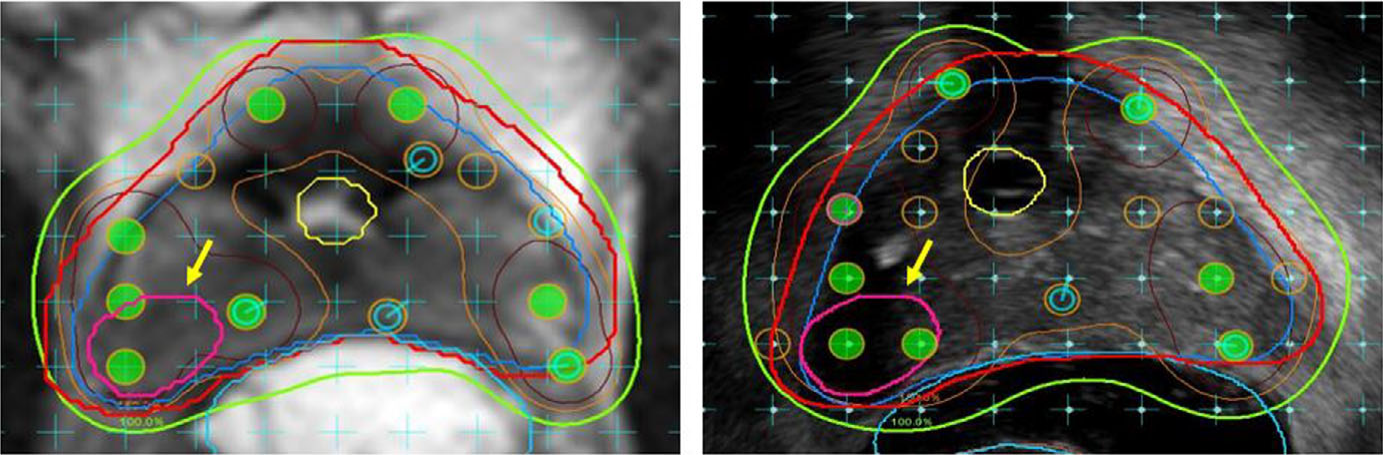
Targeted dose escalation of mpMRI‐identified PIRADS 5 lesion. Left: Preplan on T2 MRI sequence. Right: Intraoperative MRI/iTRUS fusion for real‐time planning. Contours: Royal blue, prostate; Red, PTV expansion; Yellow, Urethra; Magenta/Yellow arrow, PIRADS 5 lesion; Light blue, hydrogel spacer between prostate and rectum. Isodose lines: Green, 100% of prescription; Orange, 150%; Maroon, 200%.

In 11 (79%) cases, for which an mpMRI was obtained, a region highly suspicious for focal disease was detected and noted on final radiology read as a PIRADS 4 or 5. None of these biopsy‐proven–dominant tumor foci were retroactively seen on the corresponding pTRUS series. In all cases, the lesions were contoured in the mpMRI‐preplans as well as in the intraoperative plans and specifically targeted with dose escalation to 200% of prescription (Fig. 2). This was accomplished through intraoperative MRI/TRUS fusion and real‐time planning. All of these index lesions were located, at least in part, in the peripheral zone of the gland, and mpMRI allowed confident exclusion of any concern for extraprostatic extension. When reviewed retrospectively, as above, this distinction was not apparent on TRUS imaging, nor was accurate delineation of zonal anatomy.

**Figure 3:**
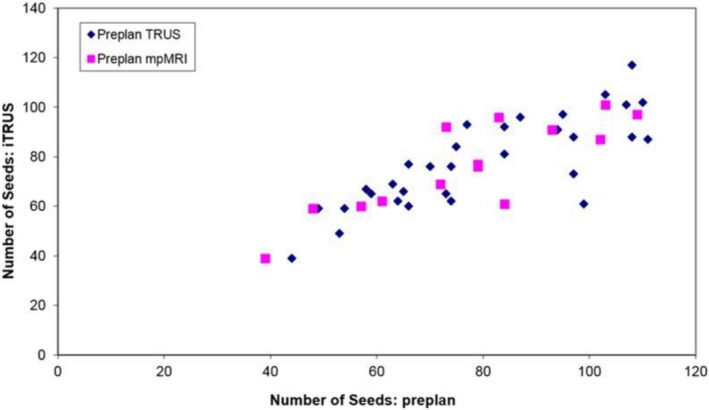
Pearson product‐moment correlation coefficient of iTRUS‐based predicted seed quantity with pTRUS (*r* = 0.87) and mpMRI (*r* = 0.86).

There was no significant difference observed in each of the modality's ability to accurately predict the number of seeds that would be required for the brachytherapy implant (Table 2). Mean differences between iTRUS vs pTRUS and iTRUS vs MRI were 7 ± 4 (*P *=* *0.92) and 5 ± 4 (*P *=* *0.31), respectively, with correlation coefficient *r* values of 0.87 and 0.86, respectively (Fig. 3). The intersample difference was nonsignificant (*P *=* *0.62). The mean percent differences between iTRUS vs pTRUS and iTRUS vs MRI were 10 ± 5% and 7 ± 7%, respectively. Predictive abilities were comparable as well in those who had received prior HT, with mean differences between iTRUS vs pTRUS and iTRUS vs MRI of 7 ± 4 (*P* = 0.31) and 3 ± 2 (*P* = 0.15), respectively. The intersample difference was similarly nonsignificant (*P *=* *0.11). The mean percent differences between iTRUS vs pTRUS and iTRUS vs MRI were 10 ± 4% and 3 ± 2%, respectively. Both imaging modalities yielded similar predictions of required intraoperative total activity, with mean differences of 7 ± 8 mCi (*P *=* *0.42) and 7 ± 8 mCi (*P *=* *0.68) for iTRUS vs pTRUS and iTRUS vs MRI, respectively, the difference between which was not significant (*P = *0.94).

**Table 2:** Seed number variation between pTRUS vs iTRUS and mpMRI vs iTRUS

Modality	Seed number range			
	0–5	6–10	11–15	16–25
pTRUS	10 (31.3%)	17 (53.1%)	3 (9.4%)	2 (6.3%)
mpMRI	9 (64.3%)	2 (14.3%)	3 (21.4%)	0 (0%)

pTRUS, preplan transrectal ultrasound; iTRUS, intraoperative transrectal ultrasound; mpMRI, multiparametric magnetic resonance imaging.

**Figure 4:**
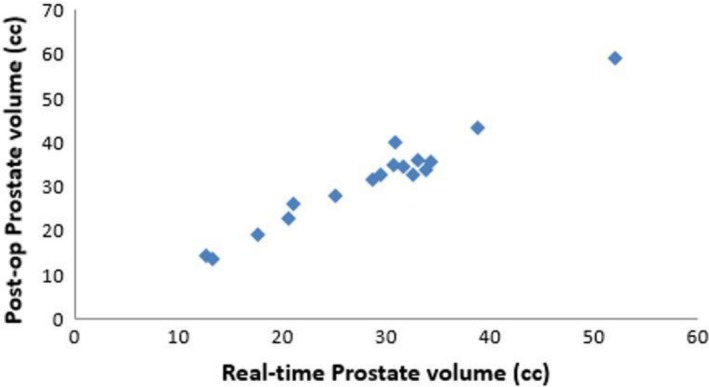
Pearson product‐moment correlation coefficient of intraoperative real‐time prostate volume (cc) with postoperative volume (*r* = 0.98).

Postoperative dosimetry was calculated from Day 0 pelvic CT in the department of radiation oncology, coregistered with iTRUS‐based and MRI‐based volumes. While prostate volume obtained with pTRUS consistently correlated with real‐time intraoperative prostate volume measurements, as well as with those on postoperative CT (*P *=* *0.19; correlation coefficient *r* = 0.98; Figure 4), there was a significant difference between intraoperative and postoperative‐based calculations of prostate V_100_ (*P *<* *0.001), V_150_ (approaching significance, *P *=* *0.08) V_200_ (*P *=* *0.03), and rectal D_1 cc_ (*P *<* *0.01), with postoperative dosimetric values higher than intraoperative measurements. No significant dosimetric difference was noted between cases utilizing ^125^I and ^103^Pd isotopes.

The surgical time for pTRUS/iTRUS cases was 79 ± 22 min, compared to 82 ± 21 min for cases where MRI fusion incorporation was performed (*P *=* *0.10). Total anesthesia time was also comparable between pTRUS alone vs mpMRI cases, 132 ± 21 min vs 139 ± 29 min (*P *=* *0.46). Times did not differ between ^125^I and ^103^Pd seed implants.

## 4 DISCUSSION

In this retrospective study of patients with low‐ and intermediate‐risk prostate cancer undergoing definitive LDR brachytherapy, we demonstrated the ability of MRI to consistently provide accurate preplanning clinical information for determining prostate volume to predict seed number and strength, equivalent to pTRUS. Furthermore, the data acquired with multiparametric MRI sequences allows for identification of regions suspicious for gross disease, facilitating enhanced dose coverage and escalation. Despite some earlier concerns reported in the literature of the potential for CT and MRI to either overestimate prostate size compared to TRUS and therefore result in overordering and wasting of seeds,^12^, ^13^ or for MRI to underestimate gland size^14^, ^15^ which could potentially lead to an inadequate number of seeds ordered, our findings support its reliability as the sole preplanning imaging modality for LDR brachytherapy, thereby obviating the need for an additional TRUS study.

While TRUS remains the most common imaging modality used for anatomic delineation of the prostate, there has been an increase in the utilization of mpMRI for staging, characterization and treatment planning of prostate cancer. Advantages of TRUS include real‐time imaging, portability, ease of use and availability. While it may allow some visualization of zonal anatomy, it is neither sensitive nor specific for detection of tumor foci, nor can it reliably detect extraprostatic extension. CT offers poor soft tissue contrast within the prostate gland and therefore very limited capability in detecting intraprostatic lesions. Some pathologic changes can be seen on CT although the location of malignant foci is hard to discern partly due to poor anatomical segmentation. Compared to these two modalities, MRI provides the highest spatial and contrast resolution of the prostate gland and surrounding soft tissues, further enhanced with increasing magnet strength. Zonal architecture is readily defined, especially on T2‐weighted sequences, as is the exact location of the urethra and seminal vesicles. Additional sequences, including DWI and DCE have the potential of detecting regions highly suspicious for tumor. While any one MRI sequence may be inadequate for radiographic diagnosis, a combination of positive findings on multiple sequence types increases the sensitivity. Given this ability to see specific highly concerning foci, the fusion of MRI to real‐time TRUS allows for high‐yield targeted biopsies to be performed, useful in planning and performing both external beam radiation treatments and brachytherapy.^16^ In the recently published PROMIS trial, Ahmed *et al*. compared the detection of clinically significant (Gleason score ≥ 4 + 3 or core length ≥ 6 mm) prostate cancer on mpMRI vs TRUS biopsy compared to a standard of template prostate mapping biopsy and found improved sensitivity with mpMRI (93% vs 48%) and 5.9% rate of serious adverse events from biopsy.^17^ The presence of extracapsular extension or seminal vesicle invasion on mpMRI (radiographic T3 disease) may be predictive for biochemical failure and distant metastasis among high‐risk patients treated with high dose rate brachytherapy and external beam radiation.^18^ In a recently published comprehensive literature review on appropriate follow up for an indeterminate lesion found on mpMRI, Gomez *et al*.^19^ offered the feasibility of close surveillance with interval mpMRI and PSA monitoring. In addition, evidence has demonstrated that MRI outperforms other imaging modalities in the detection of both large and poorly differentiated tumors.^20^ Turkbey *et al*. prospectively showed high positive predictive value (PPV) of mpMRI to detect histopathologically proven prostate cancer (98% overall), with better sensitivity for larger lesions (>5 mm) and higher Gleason score. Notably, the PPV improved with additional MRI sequences obtained beyond the standard T2 weighted sequence. Turkbey *et al*. reported the highest PPV when T2, DCE, and MR‐spectroscopy were obtained, although in the anterior peripheral zone and central gland, T2, DWI and DCE yielded significantly higher sensitivity than MR spectroscopy. Our findings corroborate the substantial advantages of obtaining a broad multiparametric analysis as opposed to the more limited added benefit of limited‐sequence MRI.

Our direct comparison of TRUS‐ vs mpMRI‐based preplanning enhances the current understanding and appreciation of advanced image‐guided brachytherapy compared to previously published experiences. Park *et al*.^12^ presented a comparison of LDR brachytherapy preplans using either CT or MRI with intraoperative TRUS and found significant differences in the predicted gland volume and required number of seeds between the different modalities. In contrast, we analyzed a cohort who underwent preplanning based on both TRUS and mpMRI, thereby directly comparing each modality's ability to predict intraoperative measurements for each unique patient, from which we concluded equivalence in the predictive abilities of mpMRI, with the additional benefits of better anatomical and pathological segmentation. Additionally, the MRIs used in that analysis were captured with 5 mm slices, while our standard is to obtain images every 3 mm, which allows for more accurate anatomic contouring of the prostate. Finally, all of the preplan MRIs obtained in our analysis included T1 and T2 weighted, and ADC mapping of DCE and DWI multiparametric analysis, not specifically mentioned in the above study.

Intraoperative MRI/TRUS fusion has emerged as an important methodology to improve accuracy in seed placement compared to TRUS alone. A recent retrospective detailed comparison of intraoperative MRI/TRUS fusion‐based dosimetry compared to postoperative CT‐based dosimetry showed superiority of MRI/TRUS to predict dose parameters to the prostate as well as exposure to the rectum.^21^ Our demonstration of the excellent predictive abilities of mpMRI serves as a critical backdrop to any discussion of intraoperative fusion‐based brachytherapy by validating the role of mpMRI‐based preplanning in place of the prior standard TRUS‐based technique, highlighting aspects in which it is at the same time equivalent to TRUS while also providing additional important guiding information.

We noted significant variation on postoperative dosimetry based on CT for prostate V_100_, V_150_, V_200_ and rectal D_1 cc_, compared to intraoperative expected values. This is most likely a result of the practice in our academic department to obtain postoperative imaging for final dosimetry on day 0 from the procedure, with the intent of providing both the most complete and educational training experience for the involved resident, as well as maximal patient convenience. If the obtained dosimetric values are within the recommended standardized national guidelines,^22^, ^23^ no further imaging is obtained. Our practice falls within the standard of obtaining postoperative imaging within 60 days of the procedure, with the majority performing it on either day 0 or 30.^23^

One concern raised with planning MRI in lieu of pTRUS is the added cost of a more advanced imaging technique, especially in the current environment of sensitivity to responsible resource stewardship. Thaker *et al*.^24^ analyzed the additional cost of MRI‐guided prostate brachytherapy compared with standard ultrasound and CT‐based brachytherapy using an innovative bottom‐up cost analysis methodology, time‐driven activity‐based costing (TDABC). As opposed to the more common approach of charge‐based accounting and fee‐for‐service which is flawed by reimbursements not serving as an accurate proxy for actual resource consumption, TDABC takes into account the personnel, equipment, and facility costs of each step in the patient's total care and calculates cost per minute for each resource consumed. They found that while MRI accounts for a relatively large portion of the total cost for brachytherapy (10%), the additional cost of an MRI‐preplanned treatment over a CT and ultrasound planned course was only 1%. The most significant cost by far is incurred in the operating room. Operating room time‐cost analyses have long showed that procedural delays and OR inefficiency adds substantial cost, calculable to the minute,^25^, ^26^ although in aggregate, compared with other surgical procedures, brachytherapy generally incurs lower OR suite costs.^27^ Importantly, our data demonstrate that the addition of intraoperative mpMRI/TRUS fusion and planning did not significantly increase total OR time. In addition, studies have demonstrated correlation between longer total anesthesia time and increased risk to the patient.^28^ We demonstrated no difference in mean anesthesia time between pTRUS‐alone–planned cases and mpMRI‐planned cases.

The application of MRI in brachytherapy treatment planning has become more routine in treatment planning of pelvic malignancies, both in the realm of gynecologic^29^ and genitourinary tumors.^8^, ^23^ Implementation of new treatment techniques is now possible due to MRI treatment planning.^30^, ^31^ A similar trend is emerging for prostate brachytherapy treatment planning. In a comprehensive review by The International Task Force on Prostate Cancer and the Focal Lesion Paradigm, the authors discussed the potential advantages as well as challenges to MRI‐guided focal prostate treatment.^32^ mpMRI image fusion can enhance the accuracy of TRUS biopsy for planning of targeted treatment approaches,^17^ as well as better detect clinically significant tumor regions.^18^, ^33^ A systematic review by Valerio *et al*., which included 2350 cases of prostate focal therapy, revealed excellent short‐ and medium‐term tumor control and incontinence and erectile dysfunction rates.^34^ Part of the impetus to investigate MRI‐based partial prostate therapy is mounting evidence that despite the potential for small regions of multifocal disease, it is the primary index lesion representing the most aggressive clonal population that carries clinical significance for potential progression and local disease recurrence.^35^, ^36^ Of course, these methods of image‐guided targeted biopsy to inform targeted treatment are imperfect and require continued investigation and improvement, an area that we continue to actively investigate. It has become our institutional practice to obtain a diagnostic/planning mpMRI prior to all prostate radiation treatment, including brachytherapy, photon and proton external beam radiation, stereotactic radiotherapy and partial prostate treatment on protocol. As illustrated in this experience (Fig. 2), any PIRADS 4 or 5 lesion identified on mpMRI is specifically contoured during preplanning for the purposes of focal dose escalation to 200% of prescription.

Limitations of our study include its retrospective nature, although in this context, with the exception of MRI‐incompatible implanted hardware, there are no patient or tumor characteristics that would lend bias to undergoing pTRUS alone vs MRI‐based preplanning. An additional limitation is the relatively modest sample size which limits our statistical power to detect statistical differences in outcome measures. The sample size, however, is comparable to that of other reported experiences in this realm, and is largely influenced by the adoption of MRI‐guided brachytherapy planning for all patients in our department. Analysis of our experience as we considered transitioning from TRUS‐based to mpMRI‐based preplanning informed the evolution of our institutional practice. Finally, some concern has been raised regarding the accuracy of TRUS/MRI fusion in the operating room due to ultrasound probe deformation of the rectum and secondarily the prostate, and that this anatomical difference may explain any variation between TRUS and MRI imaging. The MIM symphony platform accounts for these changes with fairly high fidelity, allowing for placement of a simulated virtual rectal probe at the appropriate anatomic angle, as well as rigid and deformable image registration. Real‐time TRUS guidance is applied during the brachytherapy procedure itself as a standard procedure for final assurance of quality and accuracy.

## 5 CONCLUSION

Our analysis provides further support for mpMRI‐only–based LDR prostate brachytherapy treatment preplanning, sparing the patient from an additional preplanning transrectal ultrasound. Not only does MRI predict seed number and strength with equal accuracy to pTRUS, multiparametric data carry numerous additional diagnostic, staging and preplanning advantages without substantially increasing cost to the patient. Application of mpMRI facilitates better patient selection and can allow for novel dose escalation and targeted therapy techniques. Moving forward, future studies assessing the prospective impact on treatment planning such as MRI features predicting for clinical outcome, with its impact on treatment delivery, can serve to firmly establish mpMRI as the standard of care in LDR prostate brachytherapy planning.

## CONFLICT OF INTEREST

No conflict of interest.

## DISCLOSURE

The authors report no proprietary or commercial interest in any product mentioned or concept discussed in this article.
